# Safety of Early Administration of Apixaban on Clinical Outcomes in Patients with Acute Large Vessel Occlusion

**DOI:** 10.1007/s12975-020-00839-4

**Published:** 2020-08-07

**Authors:** Shinichi Yoshimura, Kazutaka Uchida, Nobuyuki Sakai, Hirotoshi Imamura, Hiroshi Yamagami, Kanta Tanaka, Masayuki Ezura, Tadashi Nonaka, Yasushi Matsumoto, Masunari Shibata, Hajime Ohta, Masafumi Morimoto, Norihito Fukawa, Taketo Hatano, Yukiko Enomoto, Masataka Takeuchi, Takahiro Ota, Fuminori Shimizu, Naoto Kimura, Yuki Kamiya, Norito Shimamura, Takeshi Morimoto

**Affiliations:** 1 Department of Neurosurgery, Hyogo College of Medicine, Nishinomiya, Japan; 2 Department of Clinical Epidemiology, Hyogo College of Medicine, 1-1 Mukogawa, Nishinomiya, Hyogo 663-8501 Japan; 3 Department of Neurosurgery, Kobe City Medical Center General Hospital, Kobe, Japan; 4 Division of Stroke Care Unit, National Cerebral and Cardiovascular Center, Suita, Japan; 5 Department of Stroke Neurology, National Hospital Organization Osaka National Hospital, Osaka, Japan; 6 Department of Neurosurgery, National Hospital Organization Sendai Medical Center, Sendai, Japan; 7 Department of Neurosurgery, Sapporo Shiroishi Memorial Hospital, Sapporo, Japan; 8 Department of Neuroendovascular Therapy, Kohnan Hospital, Miyagi, Japan; 9 Department of Neurology, Tenri Yorozu Hospital, Nara, Japan; 10 Department of Neurosurgery, Division of Clinical Neuroscience, Faculty of Medicine, University of Miyazaki, Miyazaki, Japan; 11 Department of Neurosurgery, Yokohama Shintoshi Neurosurgical Hospital, Yokohama, Japan; 12 Department of Neurosurgery, Faculty of Medicine, Kindai University, Osaka-Sayama, Osaka Japan; 13 Department of Neurosurgery, Kokura Memorial Hospital, Fukuoka, Japan; 14 Department of Neurosurgery, Gifu University Graduate School of Medicine, Gifu, Japan; 15 Department of Neurosurgery, Seishou Hospital, Odawara, Japan; 16 Department of Neurosurgery, Tokyo Metropolitan Tama Medical Center, Tokyo, Japan; 17 Department of Neurosurgery, Shimizu Hospital, Kyoto, Japan; 18 Department of Neurosurgery, Iwate Prefectural Central Hospital, Morioka, Japan; 19 Department of Neurology, Showa University Koto Toyosu Hospital, Tokyo, Japan; 20 Department of Neurosurgery, Hirosaki University Hospital, Hirosaki, Japan

**Keywords:** Acute large vessel occlusion, Apixaban, Bleeding, Stroke

## Abstract

Early administration of direct oral anticoagulants in patients with acute large vessel occlusion (LVO) and nonvalvular atrial fibrillation (NVAF) is a concern, as endovascular therapy (EVT) became highly utilized. We conducted a historical and prospective multicenter registry at 38 centers in Japan from July 2016 to February 2018. Patients aged ≥ 20 years with NVAF and acute LVO or stenosis who received apixaban within 14 days from onset were included. We compared patients who received apixaban < 48 h (Early group) and ≥ 48 h (Late group) after onset in terms of the primary outcome (a composite of ischemic events, major bleeding events, and all-cause deaths). The secondary outcomes were each component of the primary outcome. Among the 686 patients, the median time from onset to administration was 2.5 days (range, 0–14; Early 263, Late 423). The Alberta Stroke Program Early CT Score (ASPECTS) and diffusion-weighted imaging (DWI)-ASPECTS) were significantly higher in the Early group than in the Late group. Recombinant tissue plasminogen activator (rt-PA) and EVT were more utilized in the Early group (rt-PA 46% vs. 35%, *p* = 0.003; EVT 62% vs. 46%, *p* < 0.0001). The cumulative incidence of primary outcome was similar between groups (ischemic events: Early 1.9% vs. Late 0.5% at 30 days; 3.5% vs. 0.7% at 90 days, major bleeding 3.4% vs. 2.9% at 30 days; 5.0% vs. 3.4% at 90 days). Early administration of apixaban (< 48 h), after onset of acute LVO in patients with NVAF, was generally safe compared with those who received it Late (≥ 48 h). http://www.clinicaltrials.gov. Unique identifier: NCT02818868 (June 30, 2016)

**Electronic supplementary material:**

The online version of this article (10.1007/s12975-020-00839-4) contains supplementary material, which is available to authorized users.

## Introduction

Observational studies have evaluated the efficacy of several direct oral anticoagulants (DOACs) in patients with acute ischemic stroke caused by nonvalvular atrial fibrillation (NVAF). However, the optimal timing to start anticoagulants for such patients remains unclear [1–3]. Particularly, this issue is more important for patients with acute stroke due to large vessel occlusion (LVO), a more severe form, having a higher risk for hemorrhagic changes even without anticoagulants. Such patients receive recombinant tissue plasminogen activator (rt-PA) and endovascular therapy (EVT) as the current standard therapies [4], and these interventions were associated with greater hemorrhagic complications [5, 6].

Patients with acute LVO and NVAF had higher risk of recurrence of ischemic stroke when anticoagulation was not administered appropriately [7]. Therefore, the risks of hemorrhagic complication and recurrence of ischemic stroke became challenging in patients with acute LVO and NVAF. DOACs were reported to have lower hemorrhagic complications than vitamin K antagonists [8]; however, evidence on the safety of administration of DOACs and appropriate timing in patients with acute stroke due to LVO is scarce [9].

Therefore, we had registered patients with acute LVO or intra-/extra-cranial artery stenosis and NVAF who received apixaban within 14 days after the onset and investigated the safety of early administration of apixaban for up to 1 year in real-world settings. Because a recent network meta-analysis showed that apixaban had lower incidence of major bleeding events without increment of ischemic events among all DOACs currently available in Japan [10], we registered only patients on apixaban so as not to take the differences among DOACs into account.

## Methods

We conducted a historical and prospective multicenter registry at 38 centers (Supplemental Table I) in Japan from July 2016 to February 2018. The inclusion criteria were patients aged at least 20 years, with acute ischemic stroke with LVO or intra-/extra-cranial artery stenosis and NVAF, and received apixaban within 14 days after the onset. To reflect the real-world clinical practice of LVO, we included peripheral artery occlusions such as M2-3, A1-2, or P1-2. We also included acute ischemic stroke with intra-/extra-cranial artery stenosis defined as over 50%, because anticoagulants were considered effective in patients with concomitant atherosclerotic diseases and NVAF [11, 12]. On the contrary, the exclusion criteria were patients who are considered ineligible for the study by the investigator, pregnant or potentially pregnant, have a history of hypersensitivity to apixaban, with hepatic disease having coagulation disorder and clinically important bleeding risk, with renal failure (creatinine clearance < 15 mL/min), and with pathological bleeding including intracranial bleeding of any type. The diagnostic and treatment modalities were determined by the physician-in-charge including rt-PA and EVT. The rt-PA used was alteplase, which was administered intravenously at 0.6 mg/kg [13], and EVT consisted of any type of intravascular therapy including thrombectomy using any device approved in Japan.

The institutional review boards of all 38 participating centers approved the study protocol. A written informed consent was obtained from the prospectively registered patients and from the opt-out method from the retrospectively registered patients. This procedure was approved by the institutional review boards in accordance with the Ethical Guidelines for Medical and Health Research Involving Human Subjects in Japan.

### Data Collection and Definitions

Clinical information was collected through a review of hospital charts. Follow-up information at 30, 90, and 365 days was collected by a review of hospital charts, and any additional information was collected by contacting patients, relatives, and referring physicians. We collected data on patient characteristics, modified Rankin Scale (mRS) score before the onset of stroke [14], the time from onset of symptoms to hospital arrival, baseline National Institutes of Health Stroke Scale (NIHSS), and CHA2DS2-VASc score (congestive heart failure, hypertension, age ≥ 75 years, diabetes mellitus, stroke or transient ischemic attack (TIA), vascular disease, age 65 to 74 years, and sex category) [15], the use of rt-PA, and EVT. We collected data on the spread of the infarction using the Alberta Stroke Program Early CT Score (ASPECTS) as assessed by diffusion-weighted imaging (DWI) in magnetic resonance imaging (MRI) or non-contrast computed tomography (NCCT) [16–18]. The degree of reperfusion was classified by the thrombolysis in cerebral infarction (TICI) grading system for patients with EVT [19] and modified Mori grade for the rest [5].

We also evaluated the presence of large ischemic core and intracranial hemorrhage (ICH) before apixaban administration because these factors affected the timing of apixaban administration. We used Heidelberg Bleeding Classification for the description of ICH [20]. These evaluations were conducted by the physicians in charge and were not adjudicated centrally.

### Outcomes

The primary outcome of this analysis was a composite of all-cause death, International Society on Thrombosis and Haemostasis (ISTH) major bleeding events, [21], and ischemic events. Ischemic events included ischemic stroke, acute coronary syndrome, acute myocardial infarction, or systemic embolism after apixaban administration. The secondary outcomes were each component of the primary outcome. Additionally, we assessed intracranial hemorrhage and ischemic stroke separately due to a safety concern. We analyzed these outcomes at 30, 90, and 365 days after the onset.

### Statistical Analysis

We divided all patients into those who received apixaban < 48 h after onset (Early group) and those ≥ 48 h (Late group) and assessed the differences between them. We also compared the patient characteristics between those with and without the primary outcome, and stratified patients into the Early and Late groups. The threshold of 48 h was determined by the previous report which implied that administration of DOACs within 2 days was associated with higher bleeding risk [1]. We estimated the incidences of primary and secondary outcomes from the time of apixaban administration as primary analyses. In the primary analyses, we excluded these events before apixaban administration because outcomes should occur after its administration. The secondary events after apixaban administration were included as outcomes and analyzed. We also estimated the incidences after the onset of acute LVO as sensitivity analyses. In the sensitivity analyses, all events after the onset were included.

Continuous variables are presented as mean ± standard deviation or median with interquartile range (IQR) and categorical variables as numbers and percentages. Continuous variables were compared using Student’s *t* test or the Wilcoxon rank-sum test on the basis of the distributions. Categorical variables were compared using the *χ*_2_ test when appropriate. Cumulative incidence was estimated using the Kaplan-Meier method, and the differences between the groups were assessed with the log-rank test for 365 days. The date of apixaban administration was set as the index day of the survival analyses in the primary analysis and the date of the onset of acute LVO as the index day in the sensitivity analyses. To clarify the early safety profile, we described the ischemic events before apixaban administration and tabulated the bleeding events within 30 days after apixaban administration.

The effects of the Early group relative to the Late group for primary and secondary outcomes for 365 days were estimated with Cox proportional hazard models and expressed as hazard ratios (HR) with 95% confidence intervals (CIs). We adjusted the following nine clinically relevant variables to estimate the adjusted HR in the multivariable Cox proportional hazard models: age, sex, NIHSS, rt-PA, EVT, ASPECTS, baseline glucose, creatinine, and use of antiplatelet drug before onset. We conducted additional sensitivity analysis with the Cox proportional hazard model of the primary outcome, where we replaced the baseline NIHSS and ASPECTS with the NIHSS and ASPECTS before administration of apixaban.

All statistical analyses were conducted by a physician (Uchida K) and a study statistician (Morimoto T) using JMP 14.0 (SAS Institute Inc., Cary, NC, USA). All reported *p* values were two-tailed, and *p* values < 0.05 were considered statistically significant.

## Results

### Patient Characteristics

**Fig. 1:**
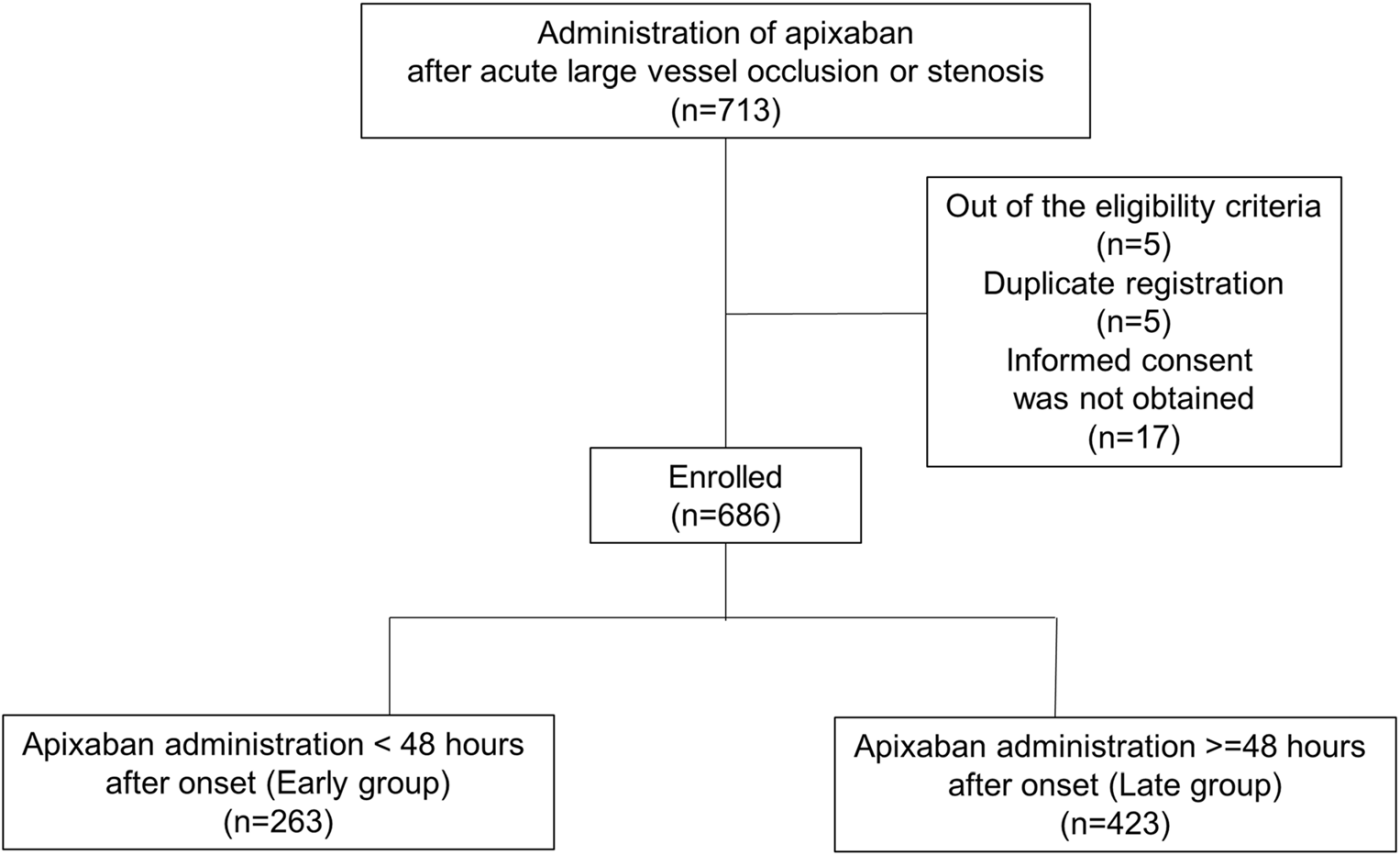
Study flowchart

We initially registered 713 patients, and 27 patients were excluded due to ineligibility, duplicate registration, and refusal to provide informed consent (Fig. 1). The median time from onset to apixaban administration was 2.5 days (IQR 1.4–5; range 0–14 days). There were 263 and 423 patients in the Early and Late groups, respectively. The mean age (SD) of enrolled patients was 77.5 (9.7) years, and men accounted for 52.0% (*n* = 357) of the patients (Table 1). Overall, 138 (20.1%) and 160 (23.3%) patients received antiplatelet and anticoagulant drugs before the onset, respectively. The rt-PA and EVT were conducted in 268 (39.1%) and 358 (52.2%) patients, respectively. Two-thirds of the patients had no disability (mRS 0) before onset.

**Table 1:** Patients’ characteristics

	All (*n* = 686)	Early group (*n* = 263)	Late group (*n* = 423)	*p* values
Age, years, mean (SD)	77.5 (9.7)	76.6 (10.5)	78.2 (9.3)	0.038
Male, *n* (%)	357 (52.0)	142 (54.0)	215 (50.8)	0.42
mRS before onset 0 or 1, *n* (%)	537/674 (79.7)	219/258 (84.9)	318/416 (76.4)	0.008
NIHSS, median (IQR)	14 (6–19)	12 (7–18)	14 (6–20)	0.17
ASPECTS, median (IQR)	8 (7–9) (*n* = 162)	9 (8–9)	8 (6–9)	0.003
DWI ASPECTS, median (IQR)	8 (6–9) (*n* = 492)	8 [7–9]	7 (6–9)	0.031
pc-ASPECTS, median (IQR)	9 (8–9) (*n* = 84)	9 (7–9)	9 (9–9)	0.91
History of cerebral infarction, *n* (%)	117 (17.1)	43 (16.3)	74 (17.5)	0.70
History of cerebral hemorrhage, *n* (%)	9 (1.3)	0 (0)	9 (2.1)	0.015
History of transient ischemic attack, *n* (%)	6 (0.9)	1 (0.4)	5 (1.2)	0.41
History of subarachnoid hemorrhage, *n* (%)	4 (0.6)	1 (0.4)	3 (0.7)	1.00
History of myocardial infarction, *n* (%)	20 (0.3)	10 (3.8)	10 (2.4)	0.28
History of unstable angina, *n* (%)	13 (1.9)	5 (1.9)	8 (1.9)	0.99
History of coronary artery disease, *n* (%)	36 (5.4)	13 (4.9)	23 (5.4)	0.77
CHA2DS2-VASc, median (IQR)	3 (2–4)	2 (2–4)	3 (2–4)	0.042
Prior antiplatelet drug, *n* (%)	138 (20.1)	53 (20.2)	85 (20.1)	0.99
Prior anticoagulant drug, *n* (%)	160 (23.3)	62 (23.6)	98 (23.2)	0.90
Warfarin, *n* (%)	90 (13.2)	39 (14.8)	51 (12.1)	0.30
DOACs, *n* (%)	69 (10.1)	23 (8.8)	46 (10.9)	0.37
Statins, *n* (%)	85 (12.4)	31 (11.8)	54 (12.8)	0.71
Occlusion, *n* (%)	642 (93.6)	253 (95.1)	389 (92.6)	0.19
Anterior circulation occlusion, *n* (%)	552 (80.5)	217 (82.5)	335 (79.2)	0.29
Internal carotid artery, *n* (%)	98 (14.3)	38 (14.4)	60 (14.2)	0.92
M1 segment middle cerebral artery, *n* (%)	231 (33.7)	82 (33.5)	149 (35.2)	0.28
M2–M3 segment middle cerebral artery, *n* (%)	228 (33.2)	98 (37.3)	130 (30.7)	0.078
A1–A2 segment anterior cerebral artery, *n* (%)	12 (1.7)	6 (2.2)	6 (1.4)	0.55
Posterior circulation occlusion, *n* (%)	91 (13.3)	35 (13.3)	56 (13.2)	0.98
Vertebral artery, *n* (%)	17 (2.5)	4 (1.5)	13 (3.1)	0.20
Basilar artery, *n* (%)	30 (4.4)	12 (4.6)	18 (4.3)	0.85
P1–P2 segment posterior cerebral artery, *n* (%)	46 (6.7)	20 (7.6)	26 (6.2)	0.46
Stenosis, *n* (%)	63 (9.2)	21 (8.0)	42 (9.9)	0.39
Anterior circulation stenosis, *n* (%)	48 (7.0)	15 (5.7)	33 (7.8)	0.29
Internal carotid artery (extra-cranial), *n* (%)	14 (2.0)	5 (1.9)	9 (2.1)	0.84
Posterior circulation stenosis, *n* (%)	20 (2.9)	8 (3.0)	12 (2.8)	1.00
Vertebral artery, *n* (%)	11 (1.6)	5 (1.9)	6 (1.4)	0.75
Basilar artery, *n* (%)	3 (0.4)	1 (0.4)	2 (0.5)	1.00
Laboratories				
Creatinine, mg/dL, median (IQR)	0.80 (0.65–1)	0.80 (0.67–1)	0.79 (0.64–0.99)	0.67
Blood glucose, mg/dL, median (IQR)	121 (105–142)	119 (106–139)	122 (105–144)	0.10
CRP, mg/dL, median (IQR)	0.15 (0.07–0.85)	0.15 (0.08–0.9)	0.15 (0.07–0.70)	0.20
PT-INR, median (IQR)	1.04 (0.98–1.14)	1.03 (0.97–1.11)	1.06 (0.99–1.16)	0.49
LDL cholesterol, mg/dL, median (IQR)	109 (89.1–130)	113 (91.3–130)	108 (88–130)	0.64
HbA1c (NGSP), %, median (IQR)	5.9 (5.6–6.2)	5.9 (5.6–6.2)	5.9 (5.6–6.2)	0.86
Initial treatment				
rt-PA, *n* (%)	268 (39.1)	121 (46.1)	147 (34.8)	0.0033
EVT, *n* (%)	358 (52.2)	163 (62.0)	195 (46.1)	< 0.0001
TICI 2b or 3, *n* (%)	329 (90.8) (*n* = 359)	152 (93.3) (*n* = 163)	178 (90.8) (*n* = 196)	0.40
Modified Mori Grade 3, *n* (%)	242 (88.6) (*n* = 273)	116 (91.3) (*n* = 127)	126 (86.3) (*n* = 146)	0.19
Intracranial hemorrhage before apixaban administration, *n* (%)	110 (16.3)	22 (8.4)	88 (20.8)	< 0.0001
Symptomatic intracranial hemorrhage, *n* (%)	0(0)	0 (0)	6 (1.4)	0.60
Heidelberg classification				
Hemorrhagic transformation of the infarcted brain tissue	84 (12.2)	18 (6.8)	66 (15.6)	0.13
Intracerebral hemorrhage within the infarcted brain tissue	6 (0.9)	0 (0)	6 (1.4)	0.087
Parenchymal hematoma remote from the infarcted brain tissue, *n* (%)	2 (0.3)	0 (0)	2 (0.5)	0.53
Intraventricular hemorrhage, *n* (%)	0 (0)	0 (0)	0 (0)	1.00
Subarachnoid hemorrhage, *n* (%)	17 (2.5)	4 (1.5)	13 (3.1)	0.31
Subdural hemorrhage, *n* (%)	1 (0.1)	0 (0)	1 (0.2)	1.00

*DWI ASPECTS*, Alberta Stroke Program Early CT Score on Diffusion-Weighted Imaging; *ASPECTS*, Alberta Stroke Program Early CT Score; *CRP*, C-reactive protein; *DOACs*, direct oral anticoagulants; *EVT*, endovascular therapy; *IQR*, interquartile range; *LDL*, low-density lipoprotein; *mRS*, modified Rankin scale; *NGSP*, National Glycohemoglobin Standardization Program; *NIHSS*, National Institute of Health Stroke Scale; *pc-ASPECTS*, posterior circulation-Alberta Stroke Program Early CT Score on Diffusion-Weighted Imaging; *PT-INR*, prothrombin time - international normalized ratio; *rt-PA*, recombinant tissue plasminogen activator; *SD*, standard deviation; *TICI*, thrombolysis in cerebral infarction grading system

Patients in the Early group were significantly younger (mean 76.6 vs. 78.2 years), had better pre-stroke mRS score, and had less severe DWI ASPECT (median 8 vs. 7) than those in the Late group. No patients in the Early group, but nine patients (2.1%) in the Late group, had a history of cerebral hemorrhage before onset. The distribution of the CHA2DS2-VASc score in the Early group was one point lower than in the Late group (median 2 vs. 3, *p* = 0.042). Other background characteristics were generally similar between the groups (Table 1). The treatment modalities for acute LVO were significantly more utilized in the Early group than in the Late group (rt-PA 46% vs. 35%; EVT 62% vs. 46%). The effective revascularization rate defined as TICI 2b or 3 for patients with EVT and modified Mori grade 3 for the rest were not different between the groups (TICI 2b or 3: 93.3% vs. 90.8%, *p* = 0.40; modified Mori grade 3: 91.3% vs. 86.3% *p* = 0.19). The ICH before apixaban administration was significantly less frequent in the Early group than in the Late group (8.4% vs. 20.8%, *p* < 0.0001).

The timing of apixaban administration was significantly later in patients with large ischemic core defined as ASPECTS, DWI ASPECTS, or pc-ASPECTS < 6 on the images before administration (Table 2). The timing of apixaban administration was also significantly later in patients with ICH than in those without ICH after EVT (median 71.7 h vs. 46.7 h, *p* = 0.001).

**Table 2:** Intervals from the onset of acute large vessel occlusions to apixaban administration by infarction volume

Imaging findings before apixaban administration	Time from onset to apixaban administration, median hours (IQR)	*p* values
ASPECTS < 6 (*n* = 54)	123.3 (56.4–181)	< 0.0001
ASPECTS ≥ 6 (*n* = 203)	61.3 (34.8–99.1)	
DWI ASPECTS < 6 (*n* = 92)	127.5 (48–173.6)	< 0.0001
DWI ASPECTS ≥ 6 (*n* = 282)	48 (30.2–92.1)	
pc-ASPECTS < 6 (*n* = 4)	194.8 (84.4–302.5)	0.004
pc-ASPECTS ≥ 6 (*n* = 59)	60 (26–120)	
With ICH (*n* = 73)	71.7 (46.9–121)	0.001
Without ICH (*n* = 286)	46.7 (29.5–96)	

*ASPECTS*, Alberta Stroke Program Early CT Score; *DWI ASPECTS*, Alberta Stroke Program Early CT Score on Diffusion-Weighted Imaging; *ICH*, intracranial hemorrhage; *pc-ASPECTS*, posterior circulation-Alberta Stroke Program Early CT Score on Diffusion-Weighted Imaging

### Primary Analyses

**Fig. 2:**
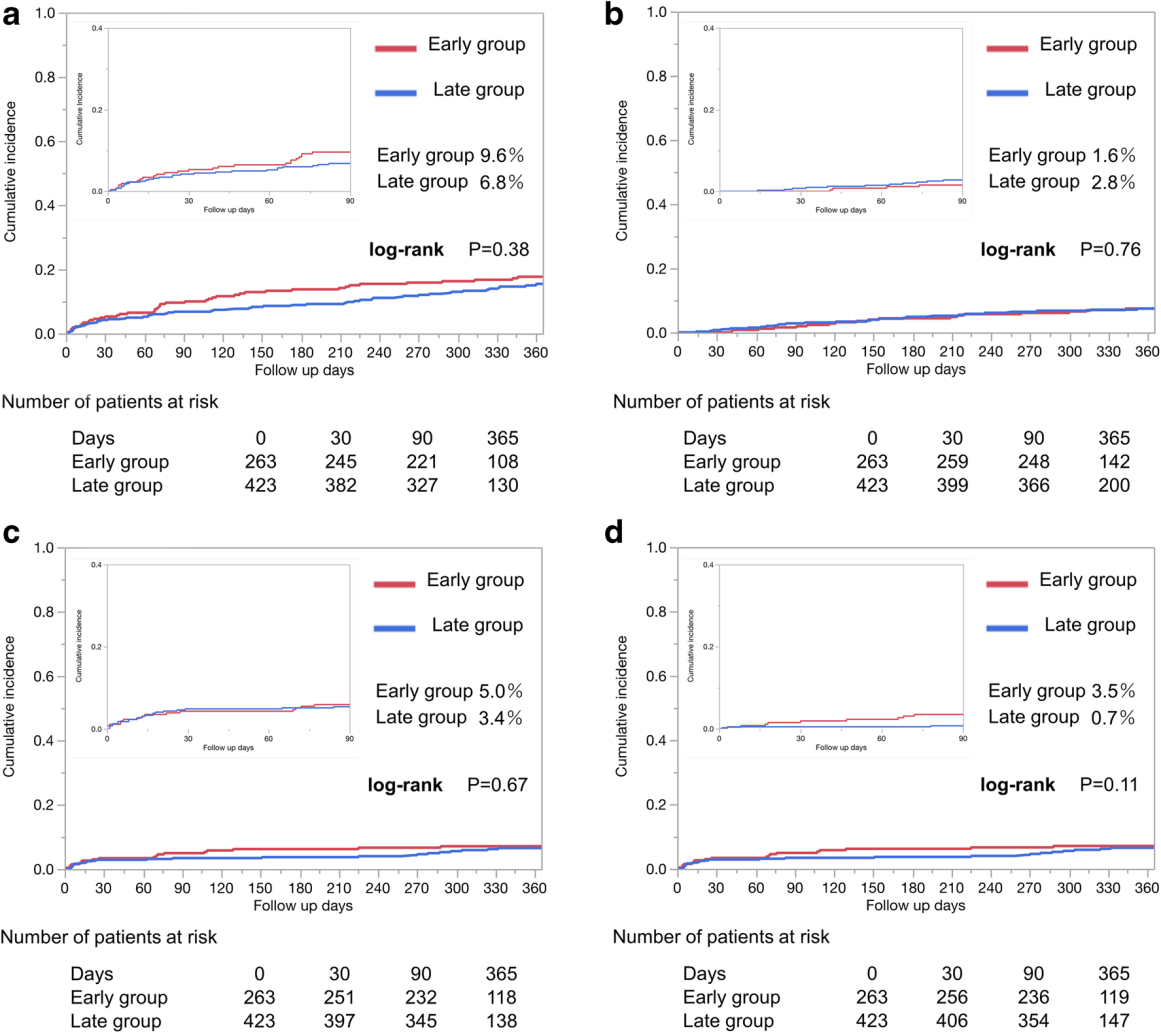
Cumulative incidences of outcomes after apixaban administration. **a** Composite event of all-cause death, ISTH major bleeding events, and recurrent ischemic events. **b** All-cause death. **c** ISTH major bleeding events. **d** Ischemic events

The cumulative incidence of primary outcome was similar between the two groups (Early 5.3% vs. Late 4.2% at 30 days; 9.6% vs. 6.8% at 90 days, 17.7% vs. 15.5% at 365 days, log-rank *p* = 0.38, Fig. 2). The crude HR of the Early group was 1.19 (95% CI 0.80–1.78) and the adjusted HR was 1.32 (95% CI 0.87–2.03) within 365 days (Table 3). Cumulative incidences of secondary outcomes including all-cause death, major bleeding events, ischemic events (all-cause death: Early 0% vs. Late 0.7% at 30 days; 1.6% vs. 2.8% at 90 days; 7.4% vs. 7.9% at 365 days, log-rank *p* = 0.76; major bleeding events: Early 3.4% vs. Late 2.9% at 30 days; 5.0% vs. 3.4% at 90 days; 7.1% vs. 6.5% at 365 days, log-rank *p* = 0.67; and ischemic events: Early 1.9% vs. Late 0.5% at 30 days; 3.5% vs. 0.7% at 90 days; 5.2% vs. 3.1% at 365 days, log-rank *p* = 0.11) were similar between the groups (Fig. 2).

**Table 3:** Outcomes of apixaban administration

	Early group (*n* = 263)			Late group (*n* = 423)			Crude HR (95% CI)	*p* values	Adjusted HR (95% CI)	*p* values
	30 days, *n* (%)	90 days, *n* (%)	365 days, *n* (%)	30 days, *n* (%)	90 days, *n* (%)	365 days, *n* (%)				
Primary outcome	14 (5.3)	25 (9.6)	44 (17.7)	17 (4.2)	27 (6.8)	54 (15.5)	1.19 (0.80–1.78)	0.38	1.32 (0.87–2.03)	0.19
All-cause death	0	4 (1.6)	18 (7.4)	3 (0.7)	11 (2.8)	27 (7.9)	0.91 (0.51–1.65)	0.76	1.22 (0.64–2.33)	0.54
ISTH major bleedings events	9 (3.4)	13 (5.0)	18 (7.1)	12 (2.9)	14 (3.4)	24 (6.5)	1.14 (0.62–2.11)	0.67	1.05 (0.56–1.99)	0.87
Intracranial hemorrhage	3 (1.1)	3 (1.1)	5 (2.0)	2 (0.5)	4 (1.0)	8 (2.3)	0.93 (0.31–2.85)	0.9	NA	NA
Ischemic events	5 (1.9)	9 (3.5)	13 (5.2)	2 (0.5)	3 (0.7)	10 (3.1)	1.95 (0.85–4.45)	0.11	NA	NA
Ischemic stroke	5 (1.9)	9 (3.5)	11 (4.3)	2 (0.5)	2 (0.5)	8 (2.5)	2.07 (0.83–5.15)	0.12	NA	NA

*CI*, confidence intervals; *HR*, hazard ratio; *NA*, not available

The patient characteristics were similar between those with and without the primary outcomes in the strata of the Early and Late groups (Supplemental Table II).

### Sensitivity Analyses

When the onset of acute LVO was set as the index day, the findings were consistent to the primary analyses (Supplemental Figure). The adjusted HR for the primary outcome was 1.21 (95% CI 0.80–1.80) and similar to that of the primary analysis.

When the NIHSS and ASPECTS values were replaced with those before apixaban administration in the primary analysis, the adjusted HR for the primary outcome was 1.64 (95% CI 0.99–2.72), and was consistent with that of the primary analysis.

### Ischemic Events Before Apixaban Administration

Ischemic events before apixaban administration occurred in three cases, which were all cerebral infarctions in the Late group. Their ASPECTS on admission were 2, 7, and 8 points. In addition, all ischemic events occurred on the 3rd day after the onset.

### Bleeding Events Within 30 Days

We tabulated the details of ISTH major bleeding events within 30 days after apixaban administration (Supplemental Table III). Eight of nine patients in the Early group and 7 of 12 patients in the Late group received rt-PA or EVT. The background of patients who developed bleeding events in 30 days was generally similar between the groups.

## Discussion

Our investigation showed that half of the physicians prescribed apixaban 2 or 3 days after the onset of acute stroke with NVAF and LVO or stenosis in daily practice. Physicians tended to prescribe apixaban early in younger and less-disabled patients before onset and with less severe DWI ASPECT. Although rt-PA or EVT had higher risk of bleeding events, such patients who received rt-PA or EVT were more likely administered apixaban at the early phase of stroke. The clinical outcomes including both bleeding and ischemic events were similar between those who received apixaban early and late, although the sample size was not large enough to confirm these findings.

The timing of apixaban administration at a median of 2.5 days in this study was much earlier than that reported in the RAF-NOACs study which showed a median of 8 days [1]. After administration of DOACs, 4.0% of the patients with acute ischemic stroke who received apixaban experienced ischemic events at 90 days in the RAF-NOACs study [1], while it was 3.5% at 90 days in patients who received apixaban within 2 days in our study. The incidence of ischemic events occurred in 2.3% of the patients with acute ischemic stroke who received DOACs at 90 days [2]. Thus, the incidence of ischemic events for acute LVO patients was similar to those of patients with acute ischemic stroke alone.

The incidence of major bleeding events at 90 days was 2.9% in patients who received apixaban after the onset of acute ischemic stroke in the RAF-NOACs study [1]. Another Japanese study indicated that major bleeding events occurred in 0.8% at 90 days in patients with acute ischemic stroke or transient ischemic attack who received rivaroxaban [2]. However, the subjects in these studies were not limited to those with acute LVO. In our study, the incidence of major bleedings at 90 days was 5.0% in patients with acute LVO who received apixaban within 2 days. Thus, the incidence of major bleedings was higher in our study than in previous reports, but these differences primarily occur because our patients experienced acute LVO, a more severe form of acute ischemic stroke.

The greatest challenge in the management of acute ischemic stroke due to LVO is the higher complication of NVAF and higher utilization rate of rt-PA and EVT. Therefore, physicians inevitably decide the appropriate timing of administration of anticoagulants. Our study enrolled consecutive patients with acute LVO and NVAF who received apixaban within 14 days and suggested that apixaban administration from the day after the onset of acute LVO was generally safe for patients from the perspective of comparable incidences of ischemic events and bleeding events with general acute stroke, even with the administration of rt-PA and EVT. Our study showed that acute LVO patients without large ischemic core or bleeding based on image evaluation of on the day after the onset received apixaban as early as possible. These findings should support the physicians on deciding the timing of initiating anticoagulant therapy for acute ischemic stroke due to LVO or stenosis. Additionally, our registry sheds light on the occurrence of ischemic strokes before apixaban administration in the Late group. Thus, apixaban could be administered as early as possible if no bleeding complication is detected on CT scans on the day of onset. Nevertheless, our findings should be confirmed by well-designed randomized clinical trials in the future.

To our knowledge, our study investigated the largest registry of patients with acute ischemic stroke due to LVO and NVAF who received DOACs in real-world settings. However, several limitations should be considered while interpreting our findings. First, this study was a registry study; thus, the timing of apixaban administration was dependent on the physicians in charge. The severity or size of each stroke changed over time, and the timing of follow-up evaluation depended on the course of the stroke. These changes were associated with the decision of administration timing and prognosis. Because this study was a multicenter registry, we did not systematically measure the infarct volumes quantitatively. We thus used the ASPECTS as a surrogate for stroke volumes. Although we adjusted the clinically relevant baseline characteristics and conducted several sensitivity analyses, the area of unadjusted confounders existed. Second, those without apixaban treatment or those who received apixaban 14 days after onset and those who were administered other DOACs were excluded. We also excluded those with chronic hepatic or renal diseases. Although apixaban is contraindicated in patients with these comorbidities, these patients sometimes received apixaban and had a higher risk of hemorrhage in the real-world setting. Therefore, there could be selection biases, and the results should be interpreted in the context that all patients received apixaban within 14 days, and did not have contraindications. Third, we systematically registered patients with acute LVO or stenosis at 38 centers, and to the best of our knowledge, this was the largest study that evaluated the safety outcome of apixaban for acute LVO. However, the sample size was still insufficient to fully evaluate the effects of apixaban on the outcomes of low incidence. For example, the incidence of ischemic events in the Early group was numerically higher than those in the Late group. This may be because the Early group had a relatively higher risk of ischemic events at the onset. Finally, this registry study was conducted in Japan. The risk for bleeding events was reported to be different between ethnicities [22]. Thus, the generalizability of our findings to the rest of the world should be carefully considered.

## Conclusions

Early administration of apixaban, less than 48 h after onset of acute stroke due to LVO in patients with NVAF, was generally safe in terms of ischemic and bleeding events. Further studies to confirm our findings should be considered for the appropriate management of acute LVO with NVAF in the future.

## Electronic Supplementary Material

ESM 1 (PDF 549 kb)
