# Correction to: Safety of Early Administration of Apixaban on Clinical Outcomes in Patients with Acute Large Vessel Occlusion

**DOI:** 10.1007/s12975-020-00871-4

**Published:** 2020-10-22

**Authors:** Shinichi Yoshimura, Kazutaka Uchida, Nobuyuki Sakai, Hirotoshi Imamura, Hiroshi Yamagami, Kanta Tanaka, Masayuki Ezura, Tadashi Nonaka, Yasushi Matsumoto, Masunari Shibata, Hajime Ohta, Masafumi Morimoto, Norihito Fukawa, Taketo Hatano, Yukiko Enomoto, Masataka Takeuchi, Takahiro Ota, Fuminori Shimizu, Naoto Kimura, Yuki Kamiya, Norito Shimamura, Takeshi Morimoto

**Affiliations:** 1 Department of Neurosurgery, Hyogo College of Medicine, Nishinomiya, Japan; 2 Department of Clinical Epidemiology, Hyogo College of Medicine, 1-1 Mukogawa, Nishinomiya, Hyogo 663-8501 Japan; 3 Department of Neurosurgery, Kobe City Medical Center General Hospital, Kobe, Japan; 4 Division of Stroke Care Unit, National Cerebral and Cardiovascular Center, Suita, Japan; 5 Department of Stroke Neurology, National Hospital Organization Osaka National Hospital, Osaka, Japan; 6 Department of Neurosurgery, National Hospital Organization Sendai Medical Center, Sendai, Japan; 7 Department of Neurosurgery, Sapporo Shiroishi Memorial Hospital, Sapporo, Japan; 8 Department of Neuroendovascular Therapy, Kohnan Hospital, Miyagi, Japan; 9 Department of Neurology, Tenri Yorozu Hospital, Nara, Japan; 10 Department of Neurosurgery, Division of Clinical Neuroscience, Faculty of Medicine, University of Miyazaki, Miyazaki, Japan; 11 Department of Neurosurgery, Yokohama Shintoshi Neurosurgical Hospital, Yokohama, Japan; 12 Department of Neurosurgery, Faculty of Medicine, Kindai University, Osaka-Sayama, Osaka, Japan; 13 Department of Neurosurgery, Kokura Memorial Hospital, Fukuoka, Japan; 14 Department of Neurosurgery, Gifu University Graduate School of Medicine, Gifu, Japan; 15 Department of Neurosurgery, Seishou Hospital, Odawara, Japan; 16 Department of Neurosurgery, Tokyo Metropolitan Tama Medical Center, Tokyo, Japan; 17 Department of Neurosurgery, Shimizu Hospital, Kyoto, Japan; 18 Department of Neurosurgery, Iwate Prefectural Central Hospital, Morioka, Japan; 19 Department of Neurology, Showa University Koto Toyosu Hospital, Tokyo, Japan; 20 Department of Neuro Surgery, Hirosaki University Hospital, Hirosaki, Japan

**Correction to: Transl. Stroke Res.**

10.1007/s12975-020-00839-4

There was an error in Fig. 2d. The correct Fig. 2d is shown below:

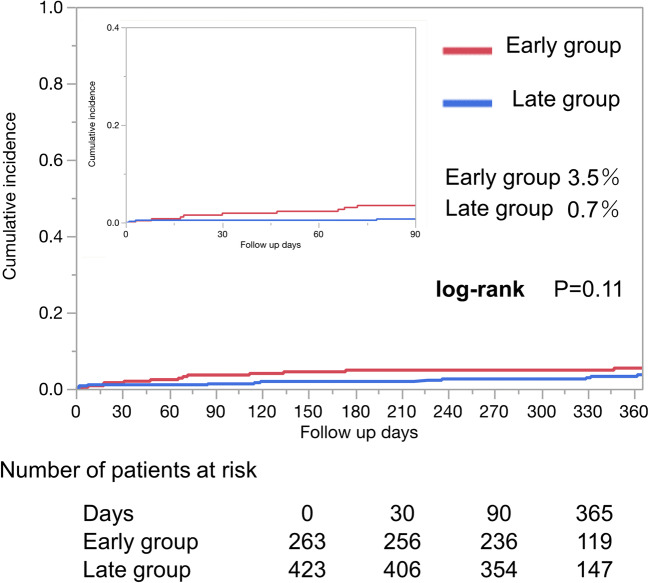



We sincerely apologize for this correction.

