# Supplementary material for: Safety of Early Administration of Apixaban on Clinical Outcomes in Patients with Acute Large Vessel Occlusion
Source: Transl Stroke Res. 2020 Aug 7;12(2):266–74. doi: 10.1007/s12975-020-00839-4 (PMC7925454; doi:10.1007/s12975-020-00839-4)
Supplement: Supplementary file 1 — (PDF 549 kb) [file 12975_2020_839_MOESM1_ESM.pdf]

SUPPLEMENTAL MATERIAL

Safety of Early Administration of Apixaban on Clinical Outcomes in Patients with Acute Large Vessel Occlusion

Supplemental Table I. Study Investigators

	Institutes	Investigators
1	Hyogo College of Medicine	Shinichi Yoshimura
2	Nishinomiya Kyouritsu Neurosurgical Hospital	Kotaro Tatebayashi
3	Seisho Hospital	Masataka Takeuchi
4	National Cerebral and Cardiovascular Center	Kazunori Toyoda
5	Iwate Prefectural Central Hospital	Naoto Kimura
6	Yoshida Hospital.Cerebrovascular Research Institute	Ikuya Yamaura
7	Sapporo Shiroishi Memorial Hospital	Tadashi Nonaka
8	Showa University Koto Toyosu Hospital	Yuki Kamiya
9	Hirosaki University	Hiroki Okuma
10	Kobe City Medical Center General Hospital	Nobuyuki Sakai
11	Yokohama Shintoshi Neurosurgical Hospital	Masafumi Morimoto
12	Goshi Hospital	Yoshiharu Oki
13	Sato Daiichi Hospital	Shigehiro Nakahara
14	Kokura Kinen Hospital	Taketo Hatano
15	Gifu University	Yukiko Enomoto
16	Fukuoka University Chikushi Hospital	Kohei Nii
17	Hakodate Shintoshi Hospital	Koichi Haraguchi
18	Kurashiki Central Hospital	Akira Handa
19	National Hospital Organization Osaka Minami Medical Center	Junya Kobayashi
20	Japan Community Health care Organinzation Kobe Central Hospital	Keigo Matsumoto
21	Mie University Hospital	Naoki Toma
22	MAZDA Hospital	Ryo Ogami
23	Shimizu Hospital	Fuminori Shimizu
24	Kyoritsu Hospital	Tomoko Iida
25	Kindai University	Amami Kato
26	Miyakonojo Medical Association Hospital	Shunro Uchinokura
27	Tokyo Metropolitan Tama Medical Center	Takahiro Ota
28	Ube-kohsan Central Hospital	Norio Ikeda
29	Japanese Red Cross Kyoto Daiichi Hospital	Keisuke Imai
30	Osaka University Hospital	Kenichi Todo

31	Kagawa University Hospital	Atsushi Shindo
32	Hirosaki Stroke and Rehabilitation Center	Joji Hagii
33	Kyoto University	Akira Ishi
34	Yamaguchi Prefectural Grand Medical Center	Hiroaki Yasuda
35	Sanda City Hospital	Akinori Nose
36	Kinki Central Hospital	Junji Ueda
37	Toranomon Hospital	Wataro Tsuruta
38	Japanese Red Cross Nagoya Daini Hospital	Keizo Yasui

Supplemental Table II. Patients' characteristics between those with and without the primary outcome

Early group (n=263)			
	With primary outcome (n=46)	Without primary outcome (n=217)	P Values
Age, years, mean (SD)	77.0 (11.9)	76.5 (10.1)	0.74
Male, n (%)	24 (52.2)	118 (54.4)	0.79
mRS before onset 0 or 1, n (%)	32/45 (71.1)	187/213 (87.8)	0.005
NIHSS, median [IQR]	16 [10-23]	12 [6-17]	0.009
ASPECTS, median [IQR]	9 [6-10] (n=9)	9 [8-9] (n=46)	0.52
DWI ASPECTS, median [IQR]	7 [6-8] (n=29)	8 [7-9] (n=160)	0.02
pc-ASPECTS, median [IQR]	9 [8-10] (n=11)	8 [7-9] (n=26)	0.03
History of cerebral infarction, n (%)	12 (14.3)	31 (26.1)	0.049
History of cerebral hemorrhage, n (%)	0 (0)	0 (0)	0
History of transient ischemic attack, n (%)	1 (2.0)	0 (0)	0.17
History of subarachnoid hemorrhage, n (%)	0 (0)	1 (0.5)	1.00
History of myocardial infarction, n (%)	3 (6.5)	7 (3.2)	0.39
History of unstable angina, n (%)	1 (2.2)	4 (1.8)	1.00
History of coronary artery disease, n (%)	1 (2.2)	12 (5.5)	0.48
CHA2DS2-VASc, median [IQR]	3 [2-4]	2 [2-3]	0.047
Prior antiplatelet drug, n (%)	14 (30.4)	39 (18.0)	0.056
Prior anticoagulant drug, n (%)	14 (30.4)	48 (22.1)	0.23
Occlusion, n (%)	45 (97.8)	205 (94.5)	0.34
Anterior circulation occlusion, n (%)	36 (78.3)	181 (83.4)	0.40
Internal carotid artery , n (%)	6 (13.0)	32 (14.8)	0.77
M1 segment middle cerebral artery, n (%)	16 (34.8)	66 (30.4)	0.56
Stenosis, n (%)	4 (8.7)	17 (7.8)	0.84
Laboratories			

Creatinine, mg/dL, median [IQR]	0.88 [0.66-1.09]	0.80 [0.67-0.97]	0.14
Blood glucose, mg/dL, median [IQR]	131 [110-151]	117 [105-136]	0.02
CRP, mg/dL, median [IQR]	0.15 [0.09-0.87]	0.15 [0.09-0.98]	0.93
PT-INR, median [IQR]	1.04 [0.96-1.16]	1.03 [0.98-1.11]	0.99
LDL cholesterol, mg/dL, median [IQR]	112 [88-140]	114 [91-129]	0.90
HbA1c (NGSP), %, median [IQR]	5.8 [5.7-6.1]	5.9 [5.6.-6.2]	0.55
Initial treatment			
rt-PA, n (%)	25 (54.4)	96 (44.2)	0.21
EVT, n (%)	34 (73.9)	129 (59.5)	0.07
TICI 2b or 3, n (%)	33 (97.1) (n=34)	119 (92.3) (n=129)	0.32
modified Mori Grade 3, n (%)	23 (92.0) (n=25)	7 (91.2) (n=102)	0.90

Late group (n=423)

	With primary outcome (n=64)	Without primary outcome (n=359)	P Values
Age, years, mean (SD)	80.3 (8.3)	77.8 (9.4)	0.053
Male, n (%)	35 (54.7)	180 (50.1)	0.50
mRS before onset 0 or 1, n (%)	48/64 (75.0)	270/352 (76.7)	0.77
NIHSS, median [IQR]	15 [5-22]	14 [6-20]	0.62
ASPECTS, median [IQR]	8 [6-9] (n=15)	8 [6-9] (n=89)	0.82
DWI ASPECTS, median [IQR]	7 [6-8] (n=44)	7 [6-9] (n=258)	0.19
pc-ASPECTS, median [IQR]	8 [7-9] (n=7)	9 [8-9] (n=42)	0.49
History of cerebral infarction, n (%)	18 (28.1)	56 (15.6)	0.020
History of cerebral hemorrhage, n (%)	1 (1.6)	8 (2.2)	1.00
History of transient ischemic attack, n (%)	0 (0)	5 (1.4)	1.00
History of subarachnoid hemorrhage, n (%)	0 (0)	3 (0.8)	1.00
History of myocardial infarction, n (%)	0 (0)	10 (2.8)	0.37
History of unstable angina, n (%)	0 (0)	8 (2.2)	0.61

History of coronary artery disease, n (%)	2 (3.1)	21 (5.9)	0.55
CHA2DS2-VASc, median [IQR]	3 [2-4]	3 [2-4]	0.03
Prior antiplatelet drug, n (%)	17 (26.6)	68 (18.9)	0.16
Prior anticoagulant drug, n (%)	18 (28.1)	80 (22.3)	0.31
Occlusion, n (%)	56(87,5)	331 (92.2)	0.21
Anterior circulation occlusion, n (%)	50 (78.1)	285 (79.4)	0.87
Internal carotid artery , n (%)	13 (20.3)	47 (13.1)	0.13
M1 segment middle cerebral artery, n (%)	19 (29.7)	130 (36.2)	0.31
Stenosis, n (%)	9 (14.1)	33 (9.2)	0.23
Laboratories			
Creatinine, mg/dL, median [IQR]	0.80 [0.62-1.08]	0.79 [0.65-0.98]	0.90
Blood glucose, mg/dL, median [IQR]	128 [110-148]	122 [105-143]	0.17
CRP, mg/dL, median [IQR]	0.17 [0.08-1.20]	0.15 [0.07-0.60]	0.32
PT-INR, median [IQR]	1.08 [1.02-1.17]	1.05 [0.98-1.15]	0.17
LDL cholesterol, mg/dL, median [IQR]	105 [80-124]	109 [89-131]	0.08
HbA1c (NGSP), %, median [IQR]	6.0 [5.7-6.5]	5.9 [5.6.-6.2]	0.13
Initial treatment			
rt-PA, n (%)	16 (25.0)	131 (36.5)	0.08
EVT, n (%)	26 (47.1)	169 (47.1)	0.34
TICI 2b or 3, n (%)	23 (88.5) (n=26)	154 (91.1) (n=169)	0.66
modified Mori Grade 3, n (%)	12 (85.7) (n=14)	114 (86.4) (n=132)	1.00

DWI ASPECTS, Alberta Stroke Program Early CT Score on Diffusion-Weighted Imaging; ASPECTS, Alberta Stroke Program Early CT Score; CRP, C-reactive protein; EVT, endovascular therapy; IQR, interquartile range; LDL, low-density lipoprotein; mRS, modified Rankin scale; NGSP, National Glycohemoglobin Standardization Program; NIHSS, National Institute of Health Stroke Scale; pc-ASPECTS, Posterior circulation-Alberta Stroke Program Early CT Score on Diffusion-Weighted Imaging; PT-INR, prothrombin time - international normalized ratio; rt-PA, recombinant tissue plasminogen activator; SD, standard deviation; TICI, thrombolysis in cerebral infarction grading system

Supplemental Table III. Bleeding events within 30 days after apixaban administration

	Age (years)	Gender	Time of administration of apixaban (day)	Time of bleeding after administration of from apixaban (day)	NIHSS	DW ASPECTS or pc-ASPECTS	Site of occlusion	Plt (10^4 /μL)	Glu (mg/dL)	PT-INR	rt-PA	EVT	Use of antiplatelet therapy before onset	Use of anticoagulant therapy before onset	Bleeding events
Early group															
1	75	Male	0	0	2	7	M1P	14	96	1.16	No	Yes	No	Yes	ICH
2	78	Male	0	10	2	7	BA	7.5	126	1	No	Yes	Yes	No	HC
3	87	Female	1	1	9	10	P2	37.5	134	1.08	Yes	No	Yes	No	Unkown
4	78	Female	1	3	25	5	M1D	21.3	95	1.08	No	Yes	No	No	GIB
5	69	Female	1	4	14	9	ICA	21.1	113	0.98	No	Yes	Yes	No	Retroperitoneal hemorrhage
6	67	Male	1	4	16	10	VA	29.7	189	1.16	Yes	Yes	No	Yes	HC
7	80	Female	1	12	19	7	M1D	20.5	135	1.09	No	Yes	No	No	GIB
8	73	Male	1	21	16	0	EICA	21.3	134	0.9	No	No	No	Yes	GIB
9	94	Female	1	26	3	7	M2	14.9	160	0.83	Yes	Yes	No	No	HC
Late group															
10	81	Female	2	12	22	6	M1P	31.6	144	0.99	No	No	Yes	No	GIB
11	85	Male	2	14	14	8	M1P	15.1	133	1.07	No	Yes	Yes	No	ICH
12	80	Female	2	18	24	8	EICA	29.8	121	1	No	Yes	No	Yes	HC
13	75	Female	3	1	23	7	BA	28.7	132	0.91	No	Yes	No	No	GIB
14	76	Male	3	4	23	9	M1P	30.3	113	1.32	No	Yes	No	Yes	HC
15	82	Male	5	2	10	9	M3	34	148	1.61	No	No	No	Yes	HC
16	59	Male	5	5	16	8	P1	22.1	106	1.13	No	No	Yes	No	HC
17	74	Male	5	6	1	9	VA	14.4	111	1.03	No	No	No	No	ICH
18	68	Female	5	16	9	4	M2	18.2	130	0.97	No	No	No	No	HC
19	82	Female	7	5	19	7	ICA	19.4	228	1.02	No	Yes	No	No	GIB
20	77	Male	12	4	7	9	P1	18.5	177	1.06	Yes	No	Yes	No	Nose bleeding
21	81	Male	12	16	5	8	Both M1P	26.9	135	1.25	Yes	Yes	No	Yes	GIB

ICA: Internal carotid artery (intracranial), EICA: Internal carotid artery (extracranial), M1P: M1 proximal, M1D: M1 distal, BA: Basilar artery, VA: Vertebral artery, ICH: Cerebral hemorrhage, HC: Hemorrhagic change after infarction, GIB: Gastrointestinal bleeding, Unknown: Unknown of bleeding source but progression of anemia.

Supplemental Figure. Cumulative incidences of outcomes after the onset of acute large vessel occlusions

A: Composite event of all-cause death, ISTH major bleedings, and ischemic events

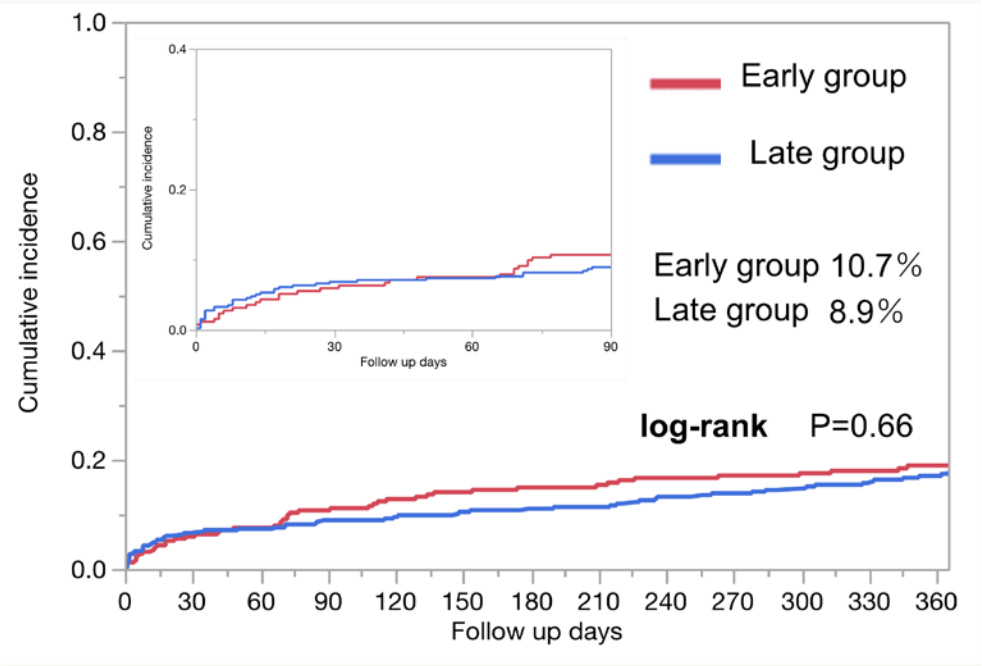

Number of patients at risk

Days	0	30	90	365
Early group	263	238	223	124
Late group	423	364	342	181

B: All-cause death

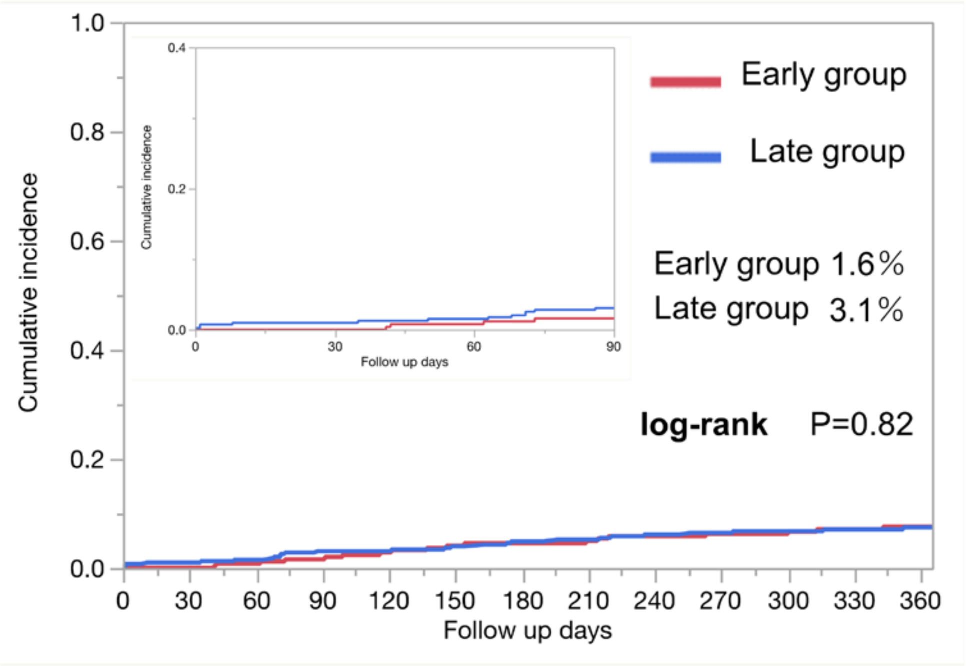

Number of patients at risk

Days	0	30	90	365
Early group	263	254	246	142
Late group	423	385	362	201

C: ISTH major bleedings

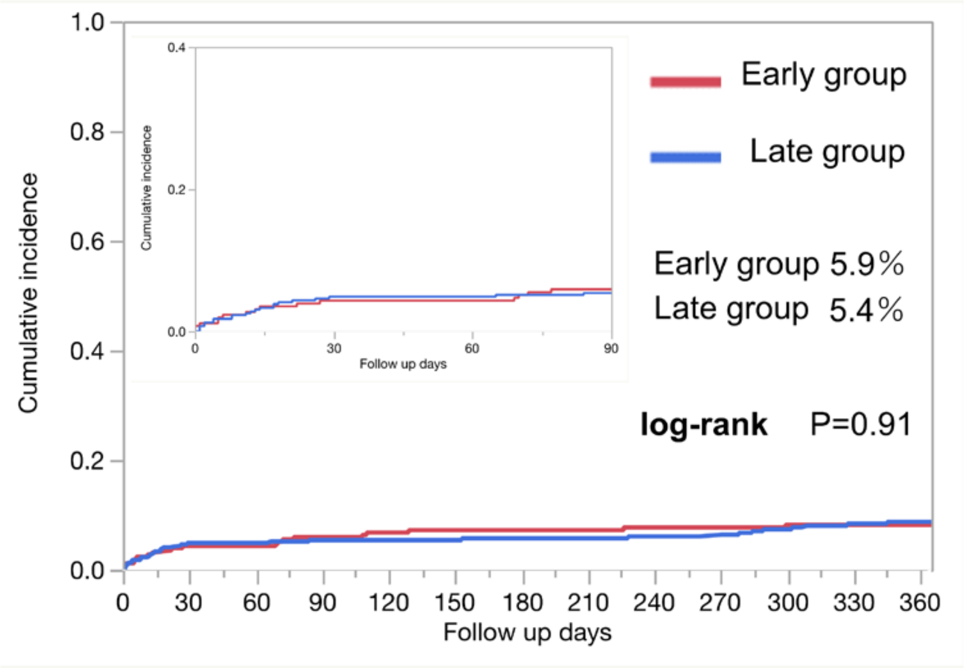

Number of patients at risk

Days	0	30	90	365
Early group	263	242	232	132
Late group	423	367	345	187

D: Ischemic events

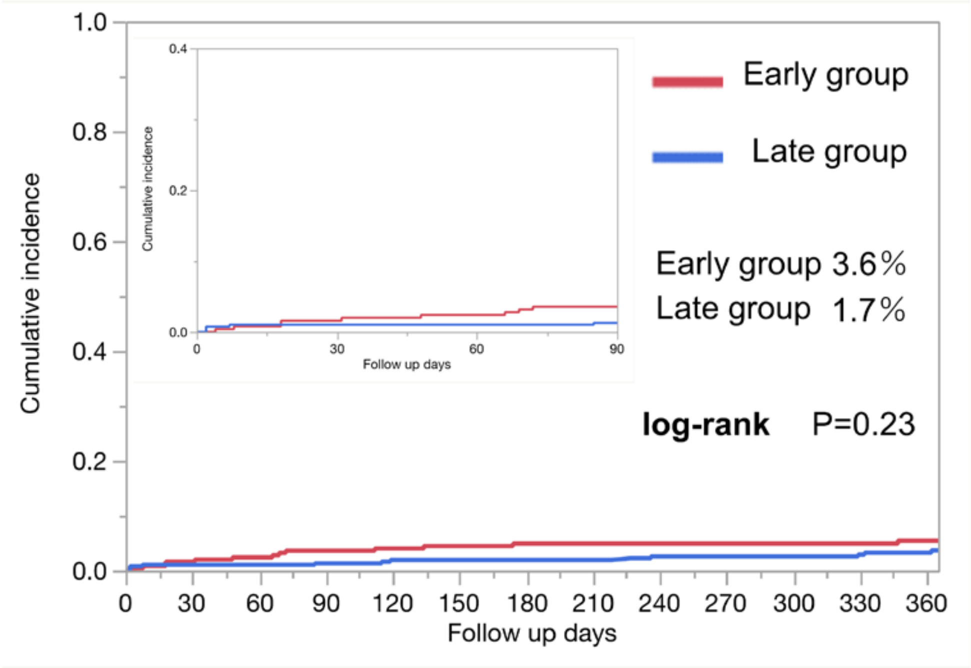

Number of patients at risk

Days	0	30	90	365
Early group	263	250	238	136
Late group	423	382	360	197
